# Competition strength influences individual preferences in an auction game

**DOI:** 10.1016/j.cognition.2014.07.010

**Published:** 2014-11

**Authors:** Ulf Toelch, Esperanza Jubera-Garcia, Zeb Kurth-Nelson, Raymond J. Dolan

**Affiliations:** a Berlin School of Mind and Brain, Humboldt University, Luisenstrasse 56, 10117 Berlin, Germany; b Wellcome Trust Centre for Neuroimaging, University College London, London WC1N 3BG, UK; c Max Planck-UCL Centre for Computational Psychiatry and Ageing, Lentzeallee 94, 14195 Berlin, Germany

**Keywords:** Social information, All pay auction, Decision-making, Preference formation, Competition

## Abstract

Competitive interactions between individuals are ubiquitous in human societies. Auctions represent an institutionalized context for these interactions, a context where individuals frequently make non-optimal decisions. In particular, competition in auctions can lead to overbidding, resulting in the so-called winner’s curse, often explained by invoking emotional arousal. In this study, we investigated an alternative possibility, namely that competitors’ bids are construed as a source of information about the good’s common value thereby influencing an individuals’ private value estimate. We tested this hypothesis by asking participants to bid in a repeated all-pay auction game for five different real items. Crucially, participants had to rank the auction items for their preference before and after the experiment. We observed a clear relation between auction dynamics and preference change. We found that low competition reduced preference while high competition increased preference. Our findings support a view that competitors’ bids in auction games are perceived as valid social signal for the common value of an item. We suggest that this influence of social information constitutes a major cause for the frequently observed deviations from optimality in auctions.

## Highlights

• We conducted a repeated all-pay auction experiment with real items.
• Participants exhibited dynamic bidding strategies.
• Bidding dynamics clearly affected preferences for auctioned items.
• Preference changes depended on the effort exerted when winning.

## 1 Introduction

Competition is integral to human social life (Festinger, 1954, Kilduff et al., 2010). It is surprising that decisions in competition contexts often deviate from rational choice even with extensive experience (Bazerman and Samuelson, 1983, Kagel and Richard, 2001, Lind and Plott, 1991). A well-studied example of such suboptimal behavior is the so-called winner’s curse in auctions where the winner often overbids the common (realizable) value of an object (Thaler, 1988). This effect has consistently been demonstrated in laboratory (Bazerman & Samuelson, 1983) and field settings (Carpenter, Holmes, & Matthews, 2008). A proposed cause for the deviation from rational choice is that individuals derive utility not only from the object itself but also from winning against competitors (for a review on further possible causes of overbidding see (Sheremeta, 2013)). This view accords with the observation that social interactions during competition elicit emotional arousal (Ku, Malhotra, & Murnighan, 2005) that individuals experience as a joy of winning respectively fear of losing (Delgado et al., 2008, Van den Bos et al., 2008).

However, apparent overbidding could also be due to an increase in the bidder’s actual preference for the good. When the true (private) value of a good is uncertain (e.g. in art auctions), competitors’ bids can be taken as information about the true value, which may drive updates to one’s own estimated value of the good. The value of a novel object is estimated by pooling previous experience with related objects (Barron, Dolan, & Behrens, 2013) but is also associated with uncertainty. By integrating over personal and social information sources, uncertainty can be reduced (Morgan et al., 2011, Rendell et al., 2011, Toelch et al., 2009). The behavior of competitors could thus serve as a proxy for the common value (Beggs and Graddy, 2009, Campbell-Meiklejohn et al., 2010, Hayes et al., 1995, Nicolle et al., 2012, Suzuki et al., 2012), particularly when uncertainty is high, social sources and social dynamics are used to update private values (Berns et al., 2010, Rendell et al., 2011, Toelch et al., 2010, Toelch et al., 2009).

Despite the recognition of competition as a social process, the interplay between competition and changes to private value estimates has received little attention. One reason is that many competition experiments are common value auctions where signals about the common value are induced (Rutström, 1998) and symmetrical (Kagel & Levin, 2008). In common value auctions, social cues (competitor bids) carry no information, a case rarely occurring under non-laboratory conditions with auctions mainly being private value auctions.

Here, we investigate an important interaction between differences in (*ex ante*) private values and the effect of subsequent competition on individuals’ (*ex post*) private value estimate. We specifically test how private values for real items are influenced by the bidding behavior in a two player multiple item repeated all-pay auction game. Crucially, we manipulated auctions such that participants encountered real competitors with lower, approximately equal, or higher private value estimates. As participants bid repeatedly and possibly opted out of the auction by bidding nothing, bids during these auctions potentially deviated from private value estimates. To account for this, we used preference^1^ statements as a proxy for participants’ private value estimates (Warren, McGraw, & Van Boven, 2011). We specifically investigated how preference ranks of the auction items changed because of both the overall level of competition and the dynamics of the auctions across the session. For this, participants ranked items by preference before and after the game. We then linked behavioral parameters from the bid progression within auctions to participants’ propensity to change their preference for a particular item.

## 2 Materials and methods

### 2.1 Participants

Participants were recruited from a local participant pool via email invitation. In total 42 (17 male) participants played the game in pairs of two with a maximum of four players per session (10 same gender pairs and 11 mixed gender pairs; sample size calculations can be found in the SI). After the experiment, participants answered a questionnaire where we collected background information like age and gender. Additionally we asked participants to give verbatim description of their strategies during the game. All procedures comply with APA guidelines and were approved by the Ethics board at Charité University hospital (EA1/212/11).

### 2.2 Auction game

Players played a first-bid all pay auction game for five different real items in pairs. Prior to playing the actual game participants received a training of 20 rounds to familiarise them with the controls and the mechanics of the game. During this training, the five auction items were replaced by abstract figures. After training, players could inspect the available auction items. All items (candle, pens, box of chocolate, one-way camera, herbal tea) were purchased at approximately the same price (4.5–5.0 Euro). The price of the items was not revealed to the participants. After inspection, players ranked the items according to their preference with 1 denoting the lowest and 5 the highest preference.

Participants played 200 auctions (40 for each item) randomly interspersed. In each round, players could distribute 100 points either to the auction item or to a monetary lottery with a price of seven Euro, which was higher than the actual cost of each item. The player with the highest amount of points allotted to the auction would win the round. The points allocated to the lottery (divided by 100) represented the chance to win seven Euro in this round. For example, take two players who bid for an item. Player 1 bids 25 points and player 2 bids 40 points. In this round player 2 wins the item and has an additional chance of 60% to win seven Euro. Player 1 does not win the auction but has a 75% chance to win the lottery. We deliberately chose a lottery as second investment options for players to minimize decision biases due to risk sensitivity. That is, allocating points in either auction or lottery entailed the risk of losing points. Overbidding in our case occurred when the sum of both players’ bids exceeded 71 (approximate value of each item: five Euro equaling 71 points). These calculations were not revealed to the participants.

At the end of the game participants had to rank the items again for preference. One round was randomly selected for each player and the outcome was paid to each participant. In other words, participants could actually win one of the items and an additional seven Euro. Participants who did not win either received three Euro alone. All participants received an additional show-up fee of five Euro. To assess participants’ private value for each item participants did not receive feedback on the outcome of the auction in the first five rounds of the experiment where all five items were presented. In all other rounds participants received feedback on whether they won the auction but not the lottery and how much the other player bid for the item.

### 2.3 Manipulation of preferences

Since we were interested in exploring the interaction between private value, social influences, and competitiveness of the environment, we performed a manipulation on the items players saw in each round by matching preferences of players in the auction. We ordered items via the preferences participants gave prior to the auction. A pair of players would bid on the item with the same preference, which was not necessarily the same item. For example, player 1 chose the candle as third preference and player 2 chose the pens as third preference so that in one round player 1 would bid for the candle while player two would bid for the pens. To create conditions with high differences between the two initial bids we also switched items of preference 2 and 4 for one of the two players in a pair. This resulted in player 1 seeing the item with the second preference and player 2 seeing the item with the fourth preference and vice versa. This effectively created three conditions where players encountered higher, equal, or lower initial bids. Players were not informed about this manipulation and remained unaware of this manipulation during the whole experiment.

### 2.4 Sample size calculation

Our sample size calculations were based on a pilot study with 10 participant pairs (*n* = 20). This study was similar in design but participants were not matched via preferences in the auctions. Pooling data from all preferences, we conducted an OLS regression with the change in the amount a participant bid over the course of an auction (dependent variable) and the initial difference between the two competitors (independent variable). In the main results, we report a similar regression that takes the multilevel structure of the data into account. For this regression, we obtained a slope of 0.58. From this, we calculated the sample size by assuming an alpha level of 0.05 and a beta level of 0.2. To detect a slope that is different from 0 with an estimated slope of 0.5 one would need more than 26 subjects. To account for possible outliers we aimed for a total number of participants between 40 and 50. Calculations were conducted with G*Power 3.1.7.

### 2.5 Analysis

For descriptive statistics, we calculated the confidence intervals via bootstrapping (10,000 iterations). For the analysis of the bidding behavior, we obtained repeated measures (bids) for each player for each item. We modeled players’ behavior via linear mixed models (package lme4 under R 3.0.2) with a random effect on the intercept for each player. We restricted our analysis to the three intermediate preference levels since we found bids of 100 and 0 frequently in the other two conditions imposing ceiling and floor effects on the bids and evolution of bids. These effects potentially distort effect estimates and associated standard errors of mixed models and with that impair inference. We selected linear mixed models based on Deviance information criterion (DIC). Our starting model consisted of all fixed effects and their respective two-way interactions. The final models were examined for patterns in the residuals (deviation from normality via QQ-plots, pattern fitted values vs. residuals).

For the analysis of preference changes, we compared the ranking of each item before and after the game that players had engaged in again limiting the analysis to the three intermediate preference levels. We modeled change as a multinomial model with *no change*, *increase*, and *decrease* of preference as dependent variables. We thus tested for the influence of factors that increased the likelihood that a player increased or decreased their preference in comparison to no change auction games. We included the preference level, the initial difference between the bids of the two players, the development of the bids compared from first to last trials, the number of wins and losses in a game, and the points that were lost during the a game as dependent variables. The latter two variables were included as they reflect competition strength between players. That is, the number of auctions a player loses is not a good indicator in itself for strong competition whereas loosing frequently in combination with loosing high amounts of points is. For the same reason a low amount of lost points will not indicate that a player won frequently. Only both variables together, even though related, give a balanced account of the competitive situation in each auction game. We also included the two-way interactions for all variables except for the preference level. We selected our final model based on the DIC. We removed interaction terms and started with effects with low effect size and wide confidence interval. We retained all interactions in the model that did not yield a reduction of DIC in the reduced model. As we collected several non-independent preference rankings for each player, we modeled player bids as a random effect on each intercept for the three preference levels. All continuous variables were z-transformed prior to fitting. We fitted the model via the MCMCglmm (Hadfield, 2010) package under R 3.0.2. We used an unspecified variance–covariance matrix for random effects and residuals allowing for unconstrained correlation in random effects and residuals. We specified priors for the residual variance as fixed. The variance for categorical dependent variables cannot be estimated since it is equal to the mean. Priors for the variance covariance for the random effect were assumed inverse Wishart distributed and parameterized as weakly informative. Final models were run for 1,000,000 iterations with a burn in of 50,000 and a thinning interval of 100. This resulted in effective sample sizes for each parameter >1000. We checked chain convergence by visually inspecting chain behavior. We further calculated the Geweke diagnostic (all values were below 2*standard error) and checked for autocorrelations within chains. Raw data and R analysis scripts are available via figshare (http://dx.doi.org/10.6084/m9.figshare.1096225).

## 3 Results

**Fig. 1:**
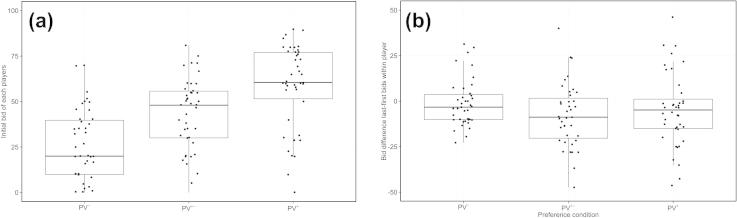
Players change their bids over the course of the experiment. (a) Players initially bid more for items with higher preferences with increasing bids for *PV*^−^; *PV^±^*; *PV^+^*. (b) Players decreased their bids during the auction in the *PV^±^* and *PV^+^* condition (see also Table 1) compared to their initial bids. Points are individual jittered data points. Boxplots show the mean and a box containing 50% of the data. Whiskers denote a 1.5 interquartile range.

Our experimental manipulation aimed at pairing participants such that they played against a player with lower, about equal, or higher private value (condition abbreviations: *PV*^+^, *PV*^±^, *PV*^−^). Because of this manipulation, the absolute difference between the initial bids of a player pair in the *PV^+^* and *PV*^−^ condition was higher than in the *PV*^±^ condition (*M_PV_*^+^_;_*_PV_*^−^ = 42.3, *95% CI* [35.8; 48.8]; *M_PV_*_±_ = 24.1, *95*% *CI* [19.1; 29.2]). Initial bids by players were ranked in the same order as preference statements and increased with preference level (Fig. 1a). Of all submitted bids players bid zero points on *M* = 14.4, 95% *CI* [8%; 21%] of all trials.

### 3.1 Dynamics during the auction

**Fig. 2:**
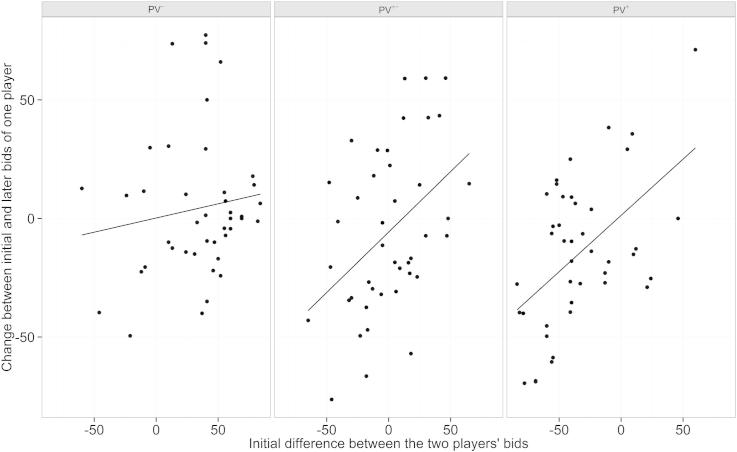
Players adjust their bid towards their competitor’s bids in the *PV*^±^ and *PV*^+^ condition. In these conditions, there is a clear relationship between the initial difference between the two player bids and the difference between the initial bid of a player and the last five bids of this player. The effect is less strong in the *PV*^−^ condition. Depicted regression lines are derived from a linear regression. For a detailed linear mixed model see Table 2.

Surprisingly, players reduced their bids over the course of auctions in the *PV*^±^ and *PV*^+^ conditions measured as the difference between the mean first five bids and the mean last five bids (Fig. 1b and Table 1). Wide confidence intervals of effect estimates (Table 1) indicate that the strength of reduction was not consistent across players. Indeed, these differences were, at least partly, driven by the initial difference between the bids of the two players in the *PV*^±^ and *PV*^+^ condition (Fig. 2). Players adjusted their bids in the direction of the bids of the other player, with stronger adjustments for the player initially bidding more (slope estimate for interactions <0.5 in Table 2). This resulted in 85% of the participants bidding initially more in the *PV*^+^ condition also winning the majority of the auctions. In the *PV*^±^ condition only 52% of the players that initially bid more also won more than half of the auctions.

**Table 1:** Linear mixed model comparing investment difference between first five and last five trials across the three preference levels. *PV^±^* and *PV^+^* contrasted with intercept (*PV*^−^).

	Estimate	95% CI
Intercept	3.64	[−3.68; 9.93]
*PV*^±^	−12.35	[−19.76; −3.71]
*PV*^+^	−11.61	[−19.32; −3.03]

**Table 2:** Linear mixed model relating the influence of preference level and difference between the initial bids in a player pair on the development of bids between the first five and last five bids of each player. The ‘:’ denotes an interaction.

	Estimate	95% CI
Intercept	−0.28	[−11.78; 10.94]
Initial Difference (ID)	0.13	[−0.11; 0.37]
*PV*^±^	−5.45	[−18.20; 7.23]
*PV*^+^	2.00	[−13.43; 17.83]
ID: *PV*^±^	0.36	[0.05; 0.67]
ID:*PV*^+^	0.35	[0.05; 0.67]

To examine the effects of underlying dynamics on a trial-to-trial basis, we focused our analysis on the effect of the two previous auction outcomes on player’s propensity to increase or decrease their bids. Player bids show a consistent pattern across all preference levels where players increased their bids when losing and decreased their bids when winning (Table 3). The positive effect on bids was slightly larger when players first won and then lost with regard to auctions with one particular item.

**Table 3:** Linear mixed model relating the change in bids between two successive rounds to the outcomes of the auctions in the two preceding rounds. LL: lose twice; LW: lose then win; WL: win then lose; WW: win twice in the previous rounds. We also explored a model with interactions between preference level and events that did not result in a decreased DIC.

	Estimate	95% CI
LL	1.93	[0.92; 2.86]
LW	−8.51	[−10.29; −6.77]
WL	7.30	[5.48; 8.94]
WW	−3.82	[5.48; 8.94]
*PV*^±^	0.69	[−0.60; 1.96]
*PV*^+^	1.62	[0.30; 2.95]

### 3.2 Preference changes

As final player bids did not reflect the preference for an item, we analyzed pre- and post-auction preference statements for the five auction items. A considerable number of players (66.6%) changed their preference ranking. Our main goal was to identify factors from the auction that influence player preference changes, an index for private value change. We found that the initial difference between player bids and the evolution of bids for a particular item affected bid dynamics (see Results on dynamics during the auction). Two additional factors entered the analysis as measures for the degree of competition: sunk costs, i.e. amount points lost in auctions, and the number of wins minus the number of losses.

**Fig. 3:**
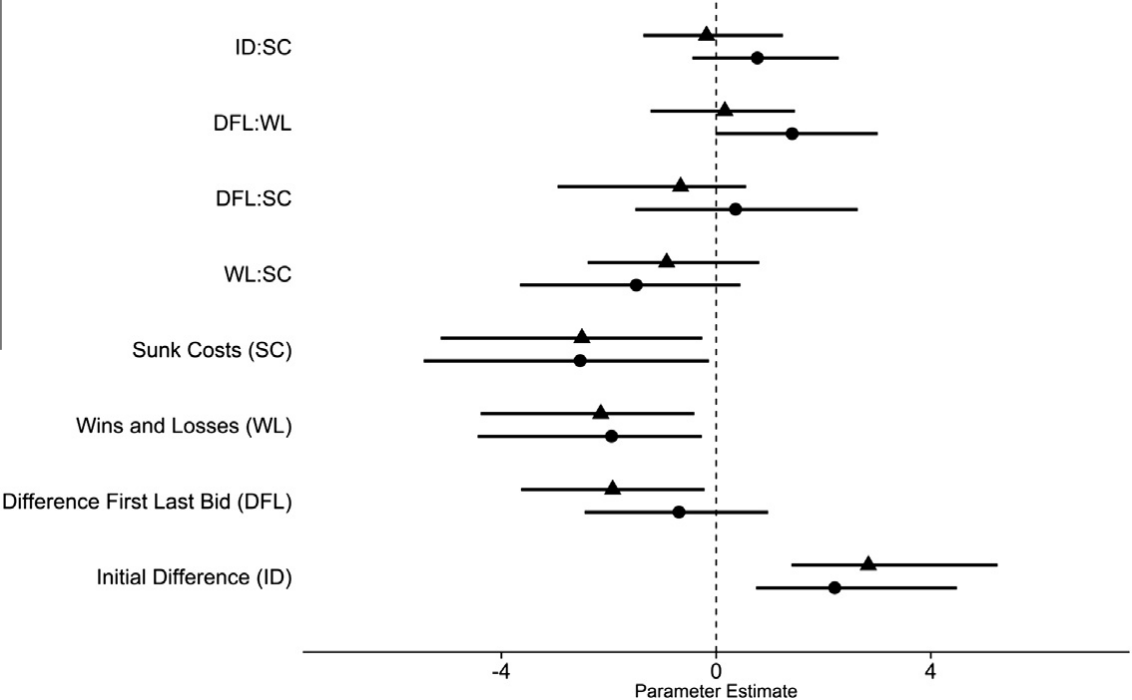
Parameter estimates (posterior means; error bars denote the 95% highest posterior density interval) of a Bayesian multinomial generalised linear mixed model. As dependent variable, we coded each auction as either increasing, decreasing, or no preference change for each player.

Based on these factors, we constructed a multinomial model where we contrasted auctions with increasing and decreasing preference with auctions without a change. Two patterns emerge from this analysis. First, some model coefficient estimates for increasing and decreasing preference point in the same direction (same sign) with approximately same effect size (Fig. 3 and Table S1). This indicates that these factors influence the probability to change preference in general, i.e. not restricted to increasing or decreasing changes. The most noteworthy of these factors was the difference between the two initial bids between the two players of a pair (ID). Here, the probability for a change increased when the competitor bid less (ID > 0; Fig. 3 and Table S1). Moreover, a high number of wins and high sunk costs (money lost in the auction) decreased the probability to change preferences.

The second pattern that emerged was characterized by factors that affected the probability to change differently (different sign or low/high parameter estimates) for increases or decreases in preference. Here, we focus on the most notable effect: the difference between the first and last bids within one player (DFL) and its interaction with the sum of wins and losses (WL). The single fixed effect of DFL is negative and twice as large for increasing as for decreasing preference changes. That is, players that increased their bids over the course of the experiment (DFL < 0) have a higher likelihood to increase their preferences (Fig. 3 and Table S1). The interaction between DFL and WL for decreasing preference changes is positive whereas the same effect for increasing preference changes is negligible. That is, players who win often and consequently decrease their bids (DFL > 0) manifest a higher likelihood of decreasing their preferences (Figs. 3 and S1).

## 4 Discussion

Our findings highlight a bidirectional influence between competitive social interactions and individuals’ preferences. We show that high competition increased preference and low competition decreased preferences. Crucially, the dynamics during the auction had a profound effect on these preference changes, which occurred mainly when participants initially bid more than their competitor. The successive evolution of bids then determined whether players increased or decreased their preference. With constant or increasing bids over the course of the auction participants increased their preference. By contrast, when competition allowed a decrease of bids, accompanied by a high number of wins throughout the auction, participants preferred this item less. That is, participants paid less than anticipated for a desired item, which resulted in a lower preference rank.

We further observed that participants did not reduce their bids to a minimum, i.e. initial value of the competitor plus some small amount. They were only able to realize a reduction from the initial difference of approximately 40–60 % towards their final bids (Fig. 2). On preference level 3 this resulted mainly from an increase in the bids of the other participant. On preference level 2 there was no significant increase of participants’ bids towards the bid of a competitor who bid for the item on preference level 4. There was, however, also no general reduction and eight participants showed an increase in bids of over 25 points (Fig. 2). One possible interpretation is that, even though this was achieved at considerable costs, participants were unwilling to surrender the item at low cost to the competitor and thus preventing a “good deal” for their opponent. Some participants reported in the post experimental questionnaire that they were actively bidding for less preferred items since it elicited positive emotions, e.g. *‘for fun I gambled for the items presumably preferred by the other player’; ‘Initially I bid according to my preferences but after a while it was more about winning’*. The strategy descriptions of the majority of players, however, are best captured by the statement of one player *‘I made choices according to the value of the item’*.

The bid dynamics we find, replicate findings from previous studies; players reduced their bids over the course of auctions (Gneezy and Smorodinsky, 2006, Sheremeta and Zhang, 2010), adjust their bids in the direction of competitor (Cason, Sheremeta, & Zhang, 2012), and increase their bids when losing and decrease their bids when winning (Kuhnen & Tymula, 2011). Over and beyond bid dynamics, our findings extend theories of decision driven preference change (Jarcho et al., 2011, Sharot et al., 2009) by showing that changes in preference are evoked by interactions between competitors. Surprisingly, winning an auction had differential effects on competitors’ private value estimates. When social information confirmed one’s private value estimate, winning resulted in an increase in private values. When social information indicated a lower item value, however, winning resulted in decreased private values. It is possible that incrementing bids (as in English auctions) might lead to an update of a bidder’s private value of an item. This seems particularly likely when uncertainty about the private value is high, e.g. art auctions, since social information will then receive a strong weight (Henrich and Boyd, 1998, Toelch et al., 2013, Toelch et al., 2014). Support for this view comes from experiments investigating repeated bidding in one shot auctions. Here, repeated feedback on the common value reduces overbidding, because trial and error learning strengthens the weight given to individual information (Dyer et al., 1989, Garvin and Kagel, 1994, Lugovskyy et al., 2010, Milgrom and Weber, 1982, Potters et al., 1998). Along the same lines, a reduction of uncertainty by the seller increases the effectiveness of the auction by reducing overbidding (Goeree & Offerman, 2003).

The findings have important implications for understanding bidding behavior in auctions. While competitive arousal (Ku et al., 2005) or the joy of winning respectively fear of losing (Bos et al., 2013, Delgado et al., 2008) can impact bidding decisions within common value auctions, we show that information derived from competitors’ bids and subsequent auction dynamics sustainably influence private value estimates. These findings suggest that individuals use social information as a proxy for the private value of an item and adjust their own private value estimate accordingly. This use of social information to reduce uncertainty has been demonstrated frequently and shown to be adaptive under a wide range of tasks (Kendal et al., 2009, Rendell et al., 2010). Individuals’ deviations from optimality predictions in auction theory thus fit a more general account that involves an evolved, and thus adaptive, psychological state in humans where social cues are weighted strongly in decision-making (Perreault et al., 2012, Toelch et al., 2013). The balance between social and personal information is then established through trial and error learning (Behrens et al., 2008, Richerson and Boyd, 2004). Common value auctions, for example, demand a reliance on individual information (estimated price and estimation error) and a neglect of competitors’ bids to bid optimally. It is thus possible that some auction experiments create environments where our proclivity to harvest social information leads to suboptimal decisions as seen in overbidding.

Several explanations have been proposed to explain overbidding in all-pay auctions (Sheremeta, 2013). Bounded rationality for example predicts that competitors increase overbidding with higher endowment. While it is possible that our per round endowment of seven Euro influenced overall overbidding rates, this explanation is not sufficient to explain the within player differences because endowments were equal across items respectively preferences. The utility of winning, as mentioned above, is also a possible cause for overbidding. While we cannot fully exclude this possibility, overbidding is happening rarely in the low preference condition. Here, only few players increase their bids over the course of the experiment. If winning an item yielded a higher utility, we again would expect similar effects across preference levels. The two aforementioned effects could potentially scale with the initial preference of the player resulting in stronger effects for high preference items. Another alternative proposed in the literature is the escalation of commitment (Staw, 1981) where competitors once committed to an action will increase their investment. The social dynamics observed in our experiment could strengthen the escalation, particular if the two competitors have similar private value estimates (as in the *PV*^±^ condition) and start overbidding each other. The escalation of commitment led to sunk costs for both players, which in turn reduced the propensity of a competitor to change their preference. Further investigations in this issue will reveal how exactly sunk costs and escalation of commitment interact with preferences.

In conclusion, our results highlight the fact that private value estimates of others, revealed through competitive interactions, contribute significantly in establishing one’s own true preferences. As preferences change frequently in our experiment, a major question that arises is how lasting these newly established preferences are. Uncovering how competitive interactions modulate general preferences, not only for single items, can further aid our understanding of human preference formation.

## Appendix A Supplementary material

Supplementary material Supplementary material contains figure and table.

## Footnotes

1 Tendency to consider something desirable or undesirable.

## References

[b0005] Barron H.C., Dolan R.J., Behrens T.E.J. (2013). Online evaluation of novel choices by simultaneous representation of multiple memories. Nature Neuroscience.

[b0010] Bazerman M.H., Samuelson W.F. (1983). I won the auction but don’t want the prize. Journal of Conflict Resolution.

[b0015] Beggs A., Graddy K. (2009). Anchoring effects: Evidence from art auctions. American Economic Review.

[b0020] Behrens T.E.J., Hunt L.T., Woolrich M.W., Rushworth M.F.S. (2008). Associative learning of social value. Nature.

[b0025] Berns G.S., Capra C.M., Moore S., Noussair C. (2010). Neural mechanisms of the influence of popularity on adolescent ratings of music. Neuroimage.

[b0030] Campbell-Meiklejohn D.K., Bach D.R., Roepstorff A., Dolan R.J., Frith C.D. (2010). How the opinion of others affects our valuation of objects. Current Biology.

[b0035] Carpenter J., Holmes J., Matthews P.H. (2008). Charity auctions: A field experiment. The Economic Journal.

[b0040] Cason T.N., Sheremeta R.M., Zhang J. (2012). Communication and efficiency in competitive coordination games. Games and Economic Behavior.

[b0045] Delgado M.R., Schotter A., Ozbay E.Y., Phelps E.A. (2008). Understanding overbidding: Using the neural circuitry of reward to design economic auctions. Science (New York, N.Y.).

[b0050] Dyer D., Kagel J.H., Levin D. (1989). A comparison of naive and experienced bidders in common value offer auctions: A laboratory analysis. The Economic Journal.

[b0055] Festinger L. (1954). A theory of social comparison processes. Human Relations.

[b0060] Garvin S., Kagel J.H. (1994). Learning in common value auctions: Some initial observations. Journal of Economic Behavior and Organization.

[b0065] Gneezy U., Smorodinsky R. (2006). All-pay auctions—an experimental study. Journal of Economic Behavior and Organization.

[b0070] Goeree J.K., Offerman T. (2003). Competitive bidding in auctions with private and common values^*^. The Economic Journal.

[b0075] Hadfield J.D. (2010). Mcmc methods for multi-response generalized linear mixed models: The mcmcglmm r package. Journal of Statistical Software.

[b0080] Hayes D.J., Shogren J.F., Shin S.Y., Kliebenstein J.B. (1995). Valuing food safety in experimental auction markets. American Journal of Agricultural Economics.

[b0085] Henrich J., Boyd R. (1998). The evolution of conformist transmission and the emergence of between-group differences. Evolution and Human Behavior.

[b0090] Jarcho J.M., Berkman E.T., Lieberman M.D. (2011). The neural basis of rationalization: Cognitive dissonance reduction during decision-making. Social Cognitive and Affective Neuroscience.

[b0095] Kagel, J. H., & Levin, D. (2008). Auctions: A survey of experimental research, 1995–2008. In *Handbook of Experimental Economics* (Vol. 2).

[b0100] Kagel J.H., Richard J.-F. (2001). Super-experienced bidders in first-price common-value auctions: Rules of thumb, nash equilibrium bidding, and the winner’s curse. Review of Economics and Statistics.

[b0105] Kendal R.L., Coolen I., Laland K.N., Dukas R., Ratcliffe J.M. (2009). Cognitive ecology II.

[b0110] Kilduff G.J., Elfenbein H.A., Staw B.M. (2010). The psychology of rivalry: A relationally dependent analysis of competition. Academy of Management Journal.

[b0115] Ku G., Malhotra D., Murnighan J.K. (2005). Towards a competitive arousal model of decision-making: A study of auction fever in live and Internet auctions. Organizational Behavior and Human Decision Processes.

[b0120] Kuhnen C.M., Tymula A. (2011). Feedback, self-esteem, and performance in organizations. Management Science.

[b0125] Lind B., Plott C.R. (1991). The winner’s curse: Experiments with buyers and with sellers. The American Economic Review.

[b0130] Lugovskyy V., Puzzello D., Tucker S. (2010). An experimental investigation of overdissipation in the all pay auction. European Economic Review.

[b0135] Milgrom P.R., Weber R.J. (1982). A theory of auctions and competitive bidding. Econometrica.

[b0140] Morgan T.J.H., Rendell L.E., Ehn M., Hoppitt W., Laland K.N. (2011). The evolutionary basis of human social learning. Proceedings of the Royal Society B: Biological Sciences.

[b0145] Nicolle A., Klein-Flügge M.C., Hunt L.T., Vlaev I., Dolan R.J., Behrens T.E.J. (2012). An agent independent axis for executed and modeled choice in medial prefrontal cortex. Neuron.

[b0150] Perreault C., Moya C., Boyd R. (2012). A Bayesian approach to the evolution of social learning. Evolution and Human Behavior.

[b0155] Potters J., de Vries C.G., van Winden F. (1998). An experimental examination of rational rent-seeking. European Journal of Political Economy.

[b0160] Rendell L., Boyd R., Cownden D., Enquist M., Eriksson K., Feldman M.W. (2010). Why copy others? Insights from the social learning strategies tournament. Science.

[b0165] Rendell L., Fogarty L., Hoppitt W.J.E., Morgan T.J.H., Webster M.M., Laland K.N. (2011). Cognitive culture: Theoretical and empirical insights into social learning strategies. Trends in Cognitive Sciences.

[b0170] Richerson P.J., Boyd R. (2004).

[b0175] Rutström Ee. (1998). Home-grown values and incentive compatible auction design. International Journal of Game Theory.

[b0180] Sharot T., Martino B.D., Dolan R.J. (2009). How choice reveals and shapes expected hedonic outcome. The Journal of Neuroscience.

[b0185] Sheremeta R.M. (2013). Overbidding and heterogeneous behavior in contest experiments. Journal of Economic Surveys.

[b0190] Sheremeta R.M., Zhang J. (2010). Can groups solve the problem of over-bidding in contests?. Social Choice and Welfare.

[b0195] Staw B.M. (1981). The escalation of commitment to a course of action. Academy of Management Review.

[b0200] Suzuki S., Harasawa N., Ueno K., Gardner J.L., Ichinohe N., Haruno M. (2012). Learning to simulate others’ decisions. Neuron.

[b0205] Thaler R.H. (1988). Anomalies: The winner’s curse. The Journal of Economic Perspectives.

[b0210] Toelch U., Bach D.R., Dolan R.J. (2013). The neural underpinnings of an optimal exploitation of social information under uncertainty. Social Cognitive and Affective Neuroscience.

[b0215] Toelch U., Bruce M.J., Meeus M.T.H., Reader S.M. (2010). Humans copy rapidly increasing choices in a multiarmed bandit problem. Evolution and Human Behavior.

[b0220] Toelch U., Bruce M.J., Newson L., Richerson P.J., Reader S.M. (2014). Individual consistency and flexibility in human social information use. Proceedings of the Royal Society B: Biological Sciences.

[b0225] Toelch U., van Delft M.J., Bruce M.J., Donders R., Meeus M.T.H., Reader S.M. (2009). Decreased environmental variability induces a bias for social information use in humans. Evolution and Human Behavior.

[b0230] Van den Bos W., Li J., Lau T., Maskin E., Cohen J.D., Montague P.R. (2008). The value of victory: Social origins of the winner’s curse in common value auctions. Judgment and Decision Making.

[b0235] van den Bos W., Talwar A., McClure S.M. (2013). Neural correlates of reinforcement learning and social preferences in competitive bidding. The Journal of Neuroscience.

[b0240] Warren C., McGraw A.P., Van Boven L. (2011). Values and preferences: Defining preference construction. Wiley Interdisciplinary Reviews: Cognitive Science.

