# Supplementary material for: Competition strength influences individual preferences in an auction game
Source: Cognition. 2014 Nov;133(2):480–7. doi: 10.1016/j.cognition.2014.07.010 (PMC4175410; doi:10.1016/j.cognition.2014.07.010)
Supplement: Supplementary material — contains figure and table. [file mmc1.pdf]

Competition strength influences individual preferences in an auction game.
Supplementary Material

Toelch et al.

Table 1: Multinomial generalized linear mixed model with logit link function corresponding to Figure 3 in the main text. Preference change served as dependent variable coded *no change*, *increase*, and *decrease*. The effect of the independent variables was estimated for increasing (left column) and decreasing (right column) preference compared to no change in preference. (Please note that due to the underlying Bayesian appraoch the 95% confidence interval is a credible interval, i.e. the probability for the true estimate being in this interval is 95% given the data and prior specification.)

	Increase			Decrease		
Parameter	l-95% CI	Post. Mode	u-95% CI	l-95% CI	Post. Mode	u-95% CI
Intercept	-3.72	-1.40	0.56	-3.89	-1.77	0.65
$PV^{\pm}$	-1.59	0.29	2.60	-2.03	-0.28	2.47
PV^{+}	-6.13	-2.42	-0.14	-4.41	-1.23	1.55
Initial Difference (ID)	1.40	2.84	5.24	0.74	2.21	4.48
Difference First Last Bid (DFL)	-3.63	-1.93	-0.22	-2.45	-0.69	0.97
Wins and Losses (WL)	-4.39	-2.15	-0.40	-4.44	-1.95	-0.27
Sunk Costs (SC)	-5.13	-2.50	-0.26	-5.45	-2.53	-0.13
WL:SC	-2.39	-0.92	0.80	-3.65	-1.49	0.45
DFL:SC	-2.95	-0.66	0.56	-1.51	0.36	2.64
DFL:WL	-1.22	0.16	1.47	0.01	1.42	3.01
ID:SC	-1.36	-0.18	1.24	-0.44	0.77	2.28

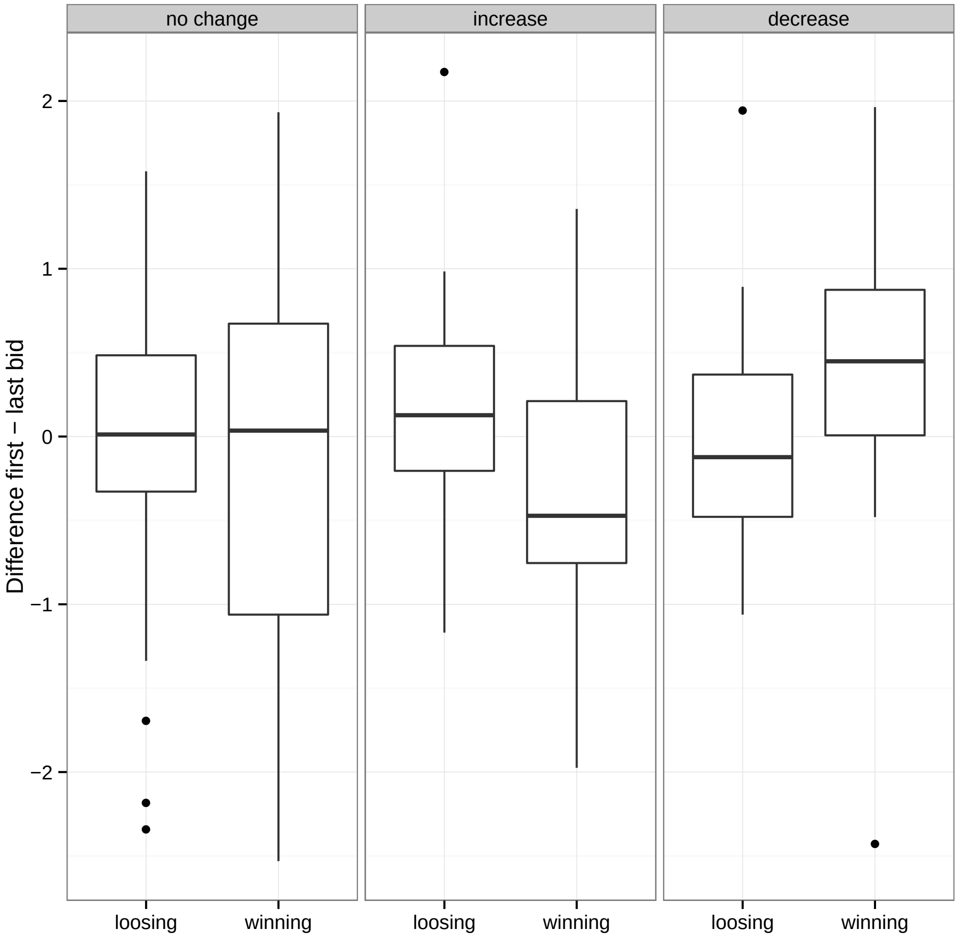

Figure 1: Players that decrease their preference for an item win often and at the same time decrease their bids during the auction. Relationship between difference between the first five and last five bids (z-transformed) and whether players were winning (more wins than losses) or loosing during the auction. The three panels refer to cases where participants did not change, increased, or decreased their preference. The depicted effect is captured in the model by the interaction DFL:WL that is reported in Figure 3 in the main text.
